# An overview of the latest developments in facial imaging

**DOI:** 10.1080/20961790.2018.1519892

**Published:** 2018-10-29

**Authors:** Carl N. Stephan, Jodi M. Caple, Pierre Guyomarc’h, Peter Claes

**Affiliations:** a Laboratory for Human Craniofacial and Skeletal Identification (HuCS-ID Lab), School of Biomedical Sciences, The University of Queensland, St Lucia, Australia;; b Unite Mixte de Recherche (UMR) 5199 De la Préhistoire à l'Actuel: Culture, Environnement et Anthropologie (PACEA), Ministère de la Culture et de la Communication (MCC), Centre National de la Recherche Scientifique (CNRS), Université de Bordeaux, Pessac, France;; c Department of Electrical Engineering, Department of Electrical Engineering (ESAT)/Processing of Speech and Images (PSI), KU Leuven, Leuven, Belgium;; d Medical Imaging Research Center (MIRC), Universitair Ziekenhuis, Leuven, Belgium

**Keywords:** Forensic anthropology, craniofacial identification, facial approximation, photographic superimposition, facial depiction, age progression, facial composites, molecular photofitting

## Abstract

Facial imaging is a term used to describe methods that use facial images to assist or facilitate human identification. This pertains to two craniofacial identification procedures that use skulls and faces—facial approximation and photographic superimposition—as well as face-only methods for age progression/regression, the construction of facial graphics from eyewitness memory (including composites and artistic sketches), facial depiction, face mapping and newly emerging methods of molecular photofitting. Given the breadth of these facial imaging techniques, it is not surprising that a broad array of subject-matter experts participate in and/or contribute to the formulation and implementation of these methods (including forensic odontologists, forensic artists, police officers, electrical engineers, anatomists, geneticists, medical image specialists, psychologists, computer graphic programmers and software developers). As they are concerned with the physical characteristics of humans, each of these facial imaging areas also falls in the domain of physical anthropology, although not all of them have been traditionally regarded as such. This too offers useful opportunities to adapt established methods in one domain to others more traditionally held to be disciplines within physical anthropology (e.g. facial approximation, craniofacial superimposition and face photo-comparison). It is important to note that most facial imaging methods are not currently used for identification but serve to assist authorities in narrowing or directing investigations such that other, more potent, methods of identification can be used (e.g. DNA). Few, if any, facial imaging approaches can be considered honed end-stage scientific methods, with major opportunities for physical anthropologists to make meaningful contributions. Some facial imaging methods have considerably stronger scientific underpinnings than others (e.g. facial approximation versus face mapping), some currently lie entirely within the artistic sphere (facial depiction), and yet others are so aspirational that realistic capacity to obtain their aims has strongly been questioned despite highly advanced technical approaches (molecular photofitting). All this makes for a broad-ranging, dynamic and energetic field that is in a constant state of flux. This manuscript provides a theoretical snapshot of the purposes of these methods, the state of science as it pertains to them, and their latest research developments.

## Introduction

Methods that assist human identification via the analysis or generation of facial graphics may be termed “facial imaging” methods (Ubelaker, personal communication 2010, unreferenced). Facial imaging thus includes facial approximation and photographic superimposition but can be further extended to age progression/regression, the construction of facial graphics from eyewitness memory (e.g. composites and sketches), facial depiction, face mapping and newly emerging methods of so–called “molecular photofitting”. Automated facial recognition systems (not considered in this review) can also justly be classified under this header.

Facial imaging methods are indispensable in many circumstances. They provide routine means of police inquiry in some instances [1,2] and valuable evidence in otherwise unsolvable cases [1,3–6]. Most of the above-mentioned methods (excluding automated facial recognition systems) achieve successful results by drawing media and public attention to the reconstructed image of a face [7,8]. This applies even if in some instances, the image of the face itself is not responsible for the successful outcome (e.g. the recognition of other items such as personal attire like ties, spectacles, hats, necklaces or shirts displayed with the face trigger recognition rather than the estimated facial morphology itself [9,10]). Subsequently, the utility of these methods has been verified in practice and often used in high-profile cases [1,3–6,9,10]. However, maximising their capabilities to generate recognition from facial morphology is beneficial. Using science to improve or test methods in the laboratory and the field is useful. As aspirational as it might be, improvements that yield any method to be so reliable that it can be used for identification as a standalone technique are ideal and should not be entirely discounted in some contexts. This offers potentially new methods and opportunities for identification and presents unique opportunities to emerging forensic anthropologists to contribute and broaden forensic anthropology input.

## Facial approximation

*Principle of the method:* to build a face based on the skull of an unidentified person so that a facial graphic can be added to, or can supplement, a media advertisement of the case [11].

*Purpose:* the face graphic acts as a point of interest to focus public attention on case details to generate additional investigative leads. At its best, the physical appearance of the face serves as the key trigger, prompting purposeful recognition of the missing person and subsequent communication of this information to the investigating authorities [12,13].

*Method:* facial approximation entails the estimation of a face from the dry skull alone. Face estimation may be executed with a variety of techniques, including (1) two-dimensional (2D) representation of the face over a photograph of the skull [14–18], (2) three-dimensional (3D) manual construction of the face in clay or mastic over the skull or skull cast [11,15,19–21], (3) computerised sculpting of the face using haptic feedback devices and a 3D scan of the skull [8,22–24], and (4) computerised construction of the face using more complex computer-automated 3D routines [25–36]. In all of these approaches, there is near-universal reliance on measurements of the thickness of the soft tissue of the face [10,11,21,24,27,37–39], which renders the commonly reported Russian–American (or Anatomical Tissue Depth) distinctions entirely obsolete [10,37–39].

*Brief history:* the first use of facial approximation in a forensic case was reported in 1916 [40,41], but the method had been studied in the context of anthropological/anatomical research since as early as 1898 [42,43]. In the early days, some anthropologists championed the forensic use of the methods [12,13,21,44–47] while others were generally more critical [43,48–50]. Computerised approaches to facial approximation were initiated in the late 1980s and early 1990s [17,18,51,52]. The term “facial approximation” has been previously used as a synonym for “facial reconstruction” [10].

*Recent research developments:* despite a long history, most methods of facial reconstruction are subjective and remain untested [53]. Many contributions have, however, been made in the last 20 years to provide quantitative and tested guidelines for soft tissue prediction leading to the delineation of facial approximation methods [10]. This includes an analysis of regional facial features, such as the position of the eyeballs in the orbits of the skull [54–65]; the position of the endo- and/or exocanthion [55,56,63,64]; the projection, width, and shape of the nose [19,21,38,66–80]; the width of the mouth [39,81–84]; the shape and size of the lips and philtrum [84,85]; the position of the eyebrows [86]; the morphology of the ears [87,88]; the morphology of large facial fat pads [89]; the morphology and thickness of the muscles of mastication [90]; and the relationship between the lines and creases of the face, and the skull [91].

The generic thickness of the envelope of the facial soft tissue around the skull has also been investigated in the last two decades: approximately 45 studies on adults [92–135] and 10 on sub-adults [92,94,109,136–143]. These studies spanned samples as diverse as Americans, Australians, Belgians, Brazilians, Canadians, Czechs, Chinese, Columbians, Egyptians, Finnish, French, Germans, Indians, Slovakians, South Koreans, Spanish and Turks. Increasing attention has been paid to the impact of measurement error in this domain, particularly where tiny differences exist due to such variables as sex [100,133,144–148]. Pooled data have been synthesised with naturally larger sample sizes (e.g. >3 200-10 300 depending on the landmark) in an effort to average out noise and triangulate on the underlying ground truths [133,143,149,150].

The correlation between the thickness of the soft tissue of the face and craniometric dimensions has been studied [96,131], with out-of-group validation tests indicating that linear regression models derived from both cadavers (needle puncture method) and living samples (B-mode ultrasound) do not out-perform mean values as point estimators [131]. In addition to arithmetic means, special cases of trimmed means have been introduced (the shorth and 75-shormax) to provide more robust central tendency estimation [145,151,152]. The establishment of a publicly accessible, free and open facial soft tissue depth data repository at CRANIOFACIALidentification.com now also provides investigators with the opportunity to conduct validation tests of any newly derived mean facial soft tissue depth using out-of-group samples [132,150].

On the technological front, advances in computer software and hardware have provided new opportunities (as applies to almost all facial imaging methods), such as core gains in fast and accurate calculations of complex algorithms for large datasets under repetitive conditions. Capabilities, even on standard desktops, are now much more advanced than they were 10 years ago. This has ushered in completely computerised and largely automated methods of facial approximation, often using CT scans as training sets [30,34–36,153–155]. Furthermore, advances in computer technology have enabled manual drawing and sculpting methods to be shifted to the electronic domain with the development of touchscreens (such as Cintiq® pen displays by WACOM® [156]) and haptic feedback devices (such as Geomagic® Touch™ by 3D SYSTEMS® [24]), respectively.

Methods for the acquisition of face shape in the research context have progressed with substantial improvements in 3D scan technology, including advances in acquisition speed, resolution, and reliability (e.g. NextEngine® by NextEngine®, Vectra® by Canfield®, 3dMDface System® by 3dMD®, DI3D® by Dimensional Imaging®, and Space Spider® by Artec®). Of course, these developments are accompanied by challenges to stay current with technology (e.g. expense), but in relative terms, the capability per dollar amount is much better now than 20 years ago and, as a result, more widely accessible. Advances in this area have enabled the comprehensive mapping of changes in soft tissue thickness with posture [148,157,158], their visualization as general trends [148], and the presentation of correction values for supine datasets, such as CT [148].

Recently developed computer-enabled tools, such as dense quasi-landmark templates, which can be automatically fitted to images of faces using iterative methods [34,36,159–162], provide powerful analytical techniques that, for example, have facilitated the estimation of face shape from genetic sequences [162,163], and have made other contributions to understanding facial growth [164] and dysmorphology [160]. Called “molecular photofitting” [165] (also see the “Molecular photofitting” section below), these methods can supplement facial approximation methods to predict faces and are especially useful for morphologies with limited tangible relationships to the skeletal structure (e.g. the colour of the iris [166–171]).

*Accuracy:* the accuracy of facial approximation, i.e. its ability to generate representative and recognisable visages of the target person (person to whom the skull belonged), should not be confused with its practical utility. As stated above, the purpose or practical utility of facial approximations in casework stretches beyond the recognition of the physical facial appearance alone to other case details independent of the face that may facilitate recognition [10,15,40,41,44,172]. Moreover, it only takes one random result of a recognition (by chance or even mistake) in a forensic case to generate a successful outcome [15,172]. In this context, the use of facial approximation to draw public attention to cases, or to act as a vehicle for recognition based on non-facial factors, often eclipses the method’s utility as a purposeful trigger for facial recognition based on face structure alone [9,10,15,44,172].

Recognition tests for facial approximations, constructed using historical methods of soft tissue prediction, have consistently displayed varied (hit-and-miss) results under controlled test conditions [15,172–177]. Even when faces are correctly recognised at rates above chance, they continue to be low (typically <35%) [15,172–177] and are well below the ceiling levels (e.g. 90-100% or higher) [174]. Improved recognition rates following the use of improved facial feature estimation methods have been recorded [65], but the face prediction toolset is rapidly expanding making most tests obsolete at the time they are conducted.

With advances in 3D technology, attempts have been made to metrically map differences between predicted 3D face surfaces and antemortem target faces by colour coding the difference on a face shell as a “heat map” [23,36]. The area of the face with a particular error can then be summarised as an overarching statement, e.g. 58% of the face surface holding an error <2.6 mm [23]. These comparisons represent an interesting line of quantitative assessment, but it should be recognized that the manner in which metric differences translate to recognition performance of human faces is likely to be complex [172,178]. Indeed, tests have shown that the perceived identity of faces does not vary simply with Euclidean distances [178]. In this context, the summary statistics of difference maps likely hold some major limits, as small metric errors with large ramifications for recognition go underemphasized, such as any small change at the orbital regions [179,180].

## Photographic superimposition

*Principle of the Method:* to compare the anatomy of a skull to an antemortem face using photographic overlay, to determine if the two are an anatomical match. This method is used in preference to facial approximation when a list of potential victims is known to the investigating authorities and antemortem facial photographs can be obtained for some or all of these individuals [181].

*Purpose:* this method has most commonly been used to exclude subjects whose face anatomy does not match that of the skull [181–186], but it has also been used, perhaps more controversially, for identification [187–189]. In certain (rare) circumstances where all ideal conditions are met (e.g. distinguishing characteristics exist, photographic conditions can be precisely replicated, image quality is high, an antemortem photograph has been obtained soon before death, and both the cranium and the mandible are present, intact and undamaged), the weight of the results of superimposition increase.

Some researchers have recently claimed that superimposition has been surpassed by other identification methods [182], but this is highly context dependent [190]. For example, in developing countries, where untracked immigration is high, obtaining DNA reference samples may be impossible. In these circumstances, the utility of facial approximation and craniofacial superimposition can, and often does, surpass DNA as an investigative approach.

*Method*: a video camera is used to record the skull, so that the original facial image and the photographic images of the skull can be aligned and superimposed (using some kind of video mixer), thereby enabling the degree of anatomical correspondence to be evaluated [181,191–193]. Ideally, the facial photographs used should be well focused and have a high resolution, should have been taken as close as possible to the time of death in the case of the facial images. It is also helpful if these photographs are multiple and represent different orientations of the subject [181,194,195]. In general, profile (lateral) facial photographs have been found to provide better comparative images than other views, but two or more views/orientations are preferred [194,195]. Owing to perspective distortion associated with the 2D rendering of 3D objects in photographs, the subject-to-camera distance should be precisely replicated [196–198]. This carries higher significance at shorter subject-to-camera distances than longer ones [196–198].

*Brief history*: like facial approximation, photographic superimposition has a long history, stretching back to its first use in casework in 1937 [199], and even earlier for academic research on the identity of historical persons [200,201]. Precursors to photographic superimposition can be traced to Welcker [202], and first concerned the superimposition of orthogonal outline tracings made using Lucae’s apparatus [203–205]. With the development of video tape recorders and cameras in 1951, the method largely moved away from still-frame photography to gain benefits in speed of skull positioning facilitated by real-time dynamic video images (but at the cost of the lower resolution in contrast to still-frame images) [191–193].

*Recent research developments*: over the past 20 years, research in craniofacial superimposition has fallen into five main domains: (1) quantification of methods’ performance using quantified landmark-based assessment [188,206–209]; (2) computer automation of methods using fuzzy logic-based approaches [210,211]—a major outcome of the New Methodologies and Protocols of Forensic Identification by the Craniofacial Superimposition Project (MEPROCS) [212]; (3) accuracy and extent of standardisation in the field [209,213,214]; (4) new means to estimate subject-to-camera distance from facial photographs [198] to account for perspective distortion recorded at the time of image capture [181,196,197,204]; and (5) improvement in anatomical assessment criteria (as outlined under the “Facial approximation” section above).

*Accuracy:* the most comprehensive study on craniofacial superimposition to date reported a false positive rate of 0.6% when both frontal and profile photographs were used [194]. However, it should be noted that the accuracy was much lower for single views (consistent fits for 9%–10% of samples, even though the vast majority of these were false matches) [194]. It is useful to note that the literature abounds in examples of superimpositions undertaken from single views, where the result has been claimed to represent a correct match, but where the outline of the skull clearly falls outside the boundaries of the anatomical soft tissue, thus indicating inconsistencies. This is highly problematic, and the risk of the use of ill-tested methods or the misapplication of methods has been amply demonstrated by the disastrous consequences of multiple incorrect identifications by superimposition, later verified by DNA [215] or radiographic comparison [216,217]. At present, there is no consensus on the accuracy of actively used methods [206,209], which is complicated by their non-standardised nature and their lack of associated validation testing. Computer science approaches promise significant advancement, but care should be taken here not to oversell emerging methods as “silver bullets”. Like all approaches, computerised solutions often hold their own limitations. Attempts should be made to verify the outcome of any craniofacial superimposition with an independent line of biological evidence to mitigate risks of error and provide robust determinations of identity.

## Age progression/regression

*Principle of the method:* to provide an image of a person’s facial appearance either prior to, or after, their last known appearance [218].

*Purpose:* age progression is undertaken when a facial photograph is available, and a facial morphology at a later or earlier chronological age (i.e. age progression and regression, respectively) is required [218]. The resulting face is often advertised publicly in the hope that the person is recognised. The utility of age progression is salient for cases of missing children, and in cases of questioned identity where recent photographs are not available in official records [218].

*Method:* age progression may be undertaken subjectively (e.g. via forensic or police art sketches [2,3,219]) or quantitatively (via age modelling software [220]). For age progression, photographs of siblings, parents, or other genetically related people may serve as guides to the final facial structure [3,218,219]. For computer-quantified methods, faces are morphed by age using average patterns of facial appearance [221–225] or more individually tailored growth trajectories—for example, delineated by regressions in principal component space [164,220,226]. Age progression/regression methods have been employed for both 2D [3,218–221] and 3D images [164,222,226]. It should be noted that age progression/regression may concern changes with growth and/or aging, depending on the starting age and the desired amount of progression/regression required.

*Brief history and recent research developments:* age progression has traditionally fallen into the domain of forensic sketch art [3,218,219]. Recent advances in computer graphics capabilities, largely concerning processing power, have enabled the development of impressive statistical approaches (see descriptions/citations above). The conservation of wrinkles, lines, and creases encoded in texture information has been one node of research focus [220,227,228], particularly as it concerns progression to more elderly faces using warps based on averages. Computer graphics-based face averaging approaches [223,229–231] have also been cross-adapted with the osteological space for the visualisation of average skull morphologies [232], as useful in physical anthropology.

*Accuracy:* single examples of age-progressed faces are common in the literature [220]. However, to the best of the authors’ knowledge, no scientific studies using facial recognition as the benchmark for accuracy have been conducted using age progression/regression as undertaken by forensic/police sketch artists. In terms of quantitative computer-assisted progression, the assessment of facial surface shells has been limited to metric assessment in 3D space (e.g. 75% of the head correctly predicted to within 3 mm [164]). As for facial approximation, it is not known how these metrics translate into human operator recognition.

## Construction of facial graphics according to eyewitness descriptions (e.g. composites and sketches)

*Principle of the method:* from an eyewitness description or account, create a facial image of a person of interest [233].

*Purpose:* the constructed facial graphic is advertised to the public in the hope that someone will recognise the face and provide new lines of inquiry [234,235]. The face is not intended to be an exact replication of the antemortem appearance, but rather a likeness good enough to be recognised [234,236]. Advertisements include “wanted” posters, either in print or on television e.g. “America’s Most Wanted” (USA), “Crimewatch” (UK) or “Crime Stoppers” (Australia).

*Method*: there are three main classes of methods used to generate faces from eyewitness descriptions: (1) forensic/police sketches using information provided in interviews [2,3,219,234,235,237]; (2) composite “kit” systems (such as Identikit*®*, Photofit*®*, COMFIT*®*, Frontalis*®,* FACES*®*, and PRO-fit*®*), where drawings or photographs of facial components are selected from facial feature libraries to construct the face either by the interviewee or the interviewer [234,235]; and (3) more advanced computer-assisted methods using “face evolution” algorithms to holistically produce face estimates (EFIT-V*®*, EvoFIT*®*) [234,235]. Note that DNA-based approaches to face prediction have also been called “facial composites” referring to the technique of photomontage or the blending of facial graphics in these methods [238]. In this paper, DNA-based methods of face prediction are addressed under their own header of “Molecular photofitting” [165], given their focus on building faces from DNA and not eyewitness reports.

*Brief History:* police sketches have the longest history in this domain [233,235]. Composite kit systems became popular in the 1960s and 1970s to by-pass the requirement for a forensic artist to sketch, thereby enabling face construction by a broader user-group of police officers [233,234]. Some of the first kits of this kind used drawings or photographs of facial features on transparency film that could be placed one on top of the other to construct the face. In the 1990s, second-generation computerised kit systems enabled many of the limits of initial systems to be overcome, such as demarcation lines between compiled features, and inability to freely adjust the size and position of facial features [233]. Computer-assisted systems that enabled holistic face estimation were first developed in 1991, and became popular around the early-to mid-2000s [235]. These programs combined faces from arrays in particular sequences (and disregarding other faces) in a process mimicking biological selection [233–235]. This class of algorithm offers the user greater ease and flexibility to create a face with the desired morphology than the adjustment of faces in a variation space along principle component vectors, such as is undertaken in other facial imaging methods (see e.g. “Age Progression” above) [235]. With each iteration of the algorithm, the user selects faces that have the greatest resemblance to that of the person in question, enabling the algorithm to “evolve” the face towards the desired result [233–235]. Some of these programs enable facial features to be locked once the desired appearance has been reached, while the rest of the face continues to be created [235]. If multiple witnesses describe a subject, the faces produced by each witness can be averaged to produce a single final face [234]. For a detailed description of the EFIT-V method, see [235]; for a more general review of multiple other methods, see [234].

*Recent research developments:* the most recent research in this area revolves around the development, testing and evaluation of feature-based and more holistic computer-assisted evolutionary programs [234,235]. Researchers have studied the impact of blurring face edges [234] and removing external facial features entirely [239,240]. The impact of interviewing strategy on accuracy rates for reconstructed faces (correct naming) has also been studied, with improvements of 15%–32% for holistic cognitive interviews [241,242]. Active caricaturing of reconstructed faces for advertisement as a video sequence is another recent development undertaken to emphasise distinctive facial features [234]. This caricaturing appears to significantly enhance recognition/naming performance [234,243]—one study reported a 10-fold increase in accuracy and another a 15% increase [234]. These video sequences of active caricaturing may be beneficial for other facial imaging methods, such as facial approximation, that thus far have not been implemented in this context (for an interactive tool to visualise changes in tissue thickness by sex, age and BMI [161]).

*Accuracy:* in the context of casework sketch methods, success heavily hinges on the quality of the initial witness interview [2,3,219,234,235,237]. It is preferable for this interview to be conducted as soon as possible after the sighting to offer the best chance for high-quality composites [234]. Sketch methods clearly also depend on the artistic talent of the sketch artist [2,3,219,237].

Frowd et al. [233,234,244,245] reported that first-generation composites had the lowest correct naming rates (1%–6%), police sketches had slightly higher ones (8%–9%) and second-generation composites had the highest (18%). It is worth mentioning that distinctive faces tend to be recognised at higher rates than undistinctive ones [246] and, as mentioned above, that recognition appears to improve by emphasising some of this distinctive information by active caricaturing [234,243]. Correct naming rates of holistically generated faces by computers is in the vicinity of 12%–55% depending on the study cited [234,247]. Fieldwork studies of the success of EFIT-V and EvoFIT, e.g. as revealed by a retrospective analysis of (300–1 000) interviews over several months, suggest a 4%–55% correct naming rate accuracy [235,248].

## Facial depiction

*Principle of the method:* to remove distracting details from postmortem facial images so that faces can be displayed in a sanitised format to family members or the public [249].

*Purpose:* to provide the family of the dead or the viewing public with sanitised facial images for recognition purposes [249]. With increases in the postmortem interval, depiction becomes more challenging but also potentially more useful as the soft tissues change from their physical antemortem appearance and become less presentable.

*Method:* postmortem facial recordings (usually photographs) serve as the basis of facial depiction, where distracting details (e.g. blood/dirt), trauma or taphonomic changes (e.g. swelling or discoloration) are removed, and a living position is provided (e.g. open eyes and closed mouth) [249,250]. Traditionally, these adjustments have been made by a forensic artist sketching the face to produce a postmortem portrait [3,219]. Computer assisted facial depiction employs image editing software such as Adobe® Photoshop® [249,250]. Often, the analyst uses a databank of facial images to extract facial features to assist in the face reconstruction process [249,250]. Thus far, postmortem portraiture and facial depiction have resided almost entirely in the forensic art sphere.

*Brief history:* postmortem portraits have been a common undertaking in the forensic art domain for many years [3,219]. The use of postmortem images, or photographs to produce sanitised images is a more recent undertaking [249,250].

*Recent research developments:* studies in this area have been limited, but one attempt (*n* = 6) quantitatively studied facial changes specifically associated with decomposition [249]. Another study of facial creases investigated their permanence in embalmed cadavers as a proxy to bloating associated with decomposition [91].

*Accuracy:* this method typically involves a large degree of subjective speculation depending on the length of the postmortem interval. No published study to date has assessed the accuracy of these methods or the utility/success of generic casework, which is most applicable to longer postmortem intervals. Note that in particular circumstances, e.g. when the postmortem interval is short and modifications do not involve the estimations of key facial features, the methods are likely more accurate [3,219,249,250]. The ability to generate realistic antemortem faces that do not appear to be a disturbing mix of antemortem and postmortem features depends entirely on the skill of the face depiction artist/analyst.

## Face mapping (photo-comparison)

*Principle of the method:* to compare a facial image(s) of an unidentified person to a reference image of a known person of interest, to determine if the unknown face corresponds to that of the reference subject [251,252].

*Purpose:* the purpose is to determine the identity of a person or, if there is no correspondence, exclude a subject of known identity from consideration. With increasing numbers of closed-circuit television and surveillance cameras, there is an increasing demand for face mapping techniques. However, differences in photographic conditions (e.g. lighting, camera angle and lens), head position, facial expression, and resolution between reference and test images are common, and severely complicate the comparisons [253].

*Method:* several methods are available for photo-comparison, which is typically achieved either by side-by-side (juxtaposed) image comparisons, or the superimposition of images—especially for 2D-to-3D comparisons in the case of superimposition. The methods can generally be grouped under the classes of: morphological analysis [251–253], photo-anthropometric analysis [251–253] and a special case of photo-anthropometric analysis called “2D facial image evaluation using 3D physiognomic data” [254–259]. Morphological analysis concerns the visual inspection of facial graphics by an analyst who draws an opinion concerning the degree of facial similarity [260]. Photo-anthropometric analysis concerns the measurement of various distances, ratios, and angles on two or more photographs to demonstrate the degree of similarity of the given faces [253]. Typically ratios and angles are used as raw distances on the two photographs can only be used when exact camera positions and subject-to-camera distances are known [251,261]. Note here that linear measurements and ratios are affected most within the same plane of rotation, e.g. in reference to the natural (upright) position of the head, the rotation of the head to one side (yaw around the *z*-axis, and thus movement in the transverse plane) affects horizontal measurements more than vertical ones [262].

Two-dimensional facial image evaluation using 3D physiognomic data was designed to solve the problems of different subject orientations between comparison images [254–259], and was first employed by Yoshino et al. [257]. In this special case of photo-anthropometric analysis, a 3D surface shell of the face of the subject of interest is acquired so that the 3D model can be rotated and positioned in the exact orientation as the subject in the 2D photograph [255,257,263]. Perspective distortion can also be matched when the subject-to-camera distance is known so that a 1:1 comparison can be undertaken [255,257,263]. The utility of this approach depends on the legal permission/freedom to acquire a 3D scan of the person of interest and the co-operation of this person during the scan acquisition process (a stationary position is normally required). The facial expression recorded in the 3D image should also be similar to that in the 2D image [263]. Between 11 and 18 points are typically marked on each image and used for metric comparisons between the 2D and the 3D conditions, which are additionally viewed on the screen for morphological inspection using custom superimposition software (such as 3D-Rugle3® by Medic Engineering®) [254,257,259,263].

*Brief history:* while the use of photographs in the courtroom has a long history (approximately 150 years in the UK, stretching back to the first use of mug shots by Bertillon [261]), facial photo-comparisons are by contrast rather recent—first used in England in 1993 and Australia in 2001 [253]. Davis et al. [261] put these dates a little earlier in the UK (1980s). Facial comparison, in the modern context, can be traced to 1993 in the literature [251]. Two-dimensional to 3D facial comparisons are more recent—2000s [254–257,259,263]—and have clear ties to methods of craniofacial superimposition [184,195,264,265].

*Recent research developments:* morphological analysis is widely regarded as a highly subjective undertaking, especially when criteria for facial comparison are far from ideal (e.g. different camera angles used). A variety of feature lists [251,266,267] and atlases [268,269] have been developed to assist these subjective morphological comparisons, but there is no universal standard. For methods of craniofacial superimposition (with which photo-comparison shares a number of similarities), the replication of camera conditions between face recording sessions, such as subject-to-camera distances, is crucial [196–198,257,261,270]. Some discussion has also addressed the applicability of qualitative scales to assess the degree of similarity and/or match in morphological analyses [271,272]. While no empirical studies have documented the accuracy of morphological facial comparisons (see the “Accuracy” section below), the Facial Identification Scientific Working Group (FISWG) recommends morphological analysis [273]. In terms of the latest quantitative research, facial image evaluation using 3D physiognomic data is the most promising, as 3D scans of subjects can be projected to the 2D plane for real-time comparison to 2D images with controls concerning the camera’s position [257,259]. This enables a replication of perspective distortion in all its forms to achieve one-to-one comparisons with impressive visual and metric support [254–257]. However, as reviewed in the “Accuracy” section below, even this method of photo-comparison has limits, and is not free of controversy [259,261,273,274].

*Accuracy:* the science underlying the validation of face mapping protocols as employed by human operators to evaluate 2D face images in juxtaposed or superimposed positions without repeated photography conditions is, in general, weak and incomplete [262]. Ideally, for any photo-comparison, the facial images to be compared should be high quality, the time between instances of image acquisition should be negligible/minimal and the photographic conditions under which the compared images are acquired should be known and identical (e.g. same lighting, camera angle, subject-to-camera distance, head position and facial expression). Rarely, however, are these requirements met in practice, thus adding complexity to facial comparisons conducted in real-life forensic casework [253,261,262,273].

It is important to note that changes in any one particular criterion does not necessarily invalidate comparisons, depending on the circumstance and the extent of the departure from the ideal. For example, while subject-to-camera distances affect how a 3D object is projected to a 2D plane, it is equally important to note that the degree of perspective distortion is differential [196–198,270]. That is, the impact is considerably greater at shorter subject-to-camera distances than longer ones [196–198,270]. Therefore, depending on the extent of the conditions applicable, comparisons may or may not be valid. It is also important to note that it is not the lens of the camera that introduces the perspective distortion, as is still erroneously assumed at times [253,261], but rather the distance at which the lens is placed [181,196–198,275]. Thus, the lens does not produce the perspective distortion: the subject-to-camera distance does. Short focal length lenses simply permit closer subject-to-camera distances without parts of the subject being outside the field of view [275]. The lens may be responsible for other aberrations, such as imperfections toward its edges [275].

So far as quantitative tests of 2D-to-2D photo-morphometric methods are concerned, the results have not been positive [276–278]. For example, Moreton and Morely [277] found variability in facial measurements due to differences in camera angle, to be as great as those in facial measurements between subjects. Kleinberg et al. [276] also found that the ectocanthion, nasion and stomion landmarks failed to accurately identify targets using line-ups of 10 subjects (selected from a pool of 120 subjects and presented as 80 groups). Data such as these have led to notable bodies, such as the FISWG 2012, advising against the use of photo-anthropometry for image comparison [253,273]. Further, the use of landmarks in 3D [278–280] and 2D images [279–281] has yielded substantial inaccuracies, in part owing to a lack of reliability for human operator placed landmarks, but also because of the misappropriation of 3D landmarks to 2D contexts [280]. While the superimposition technique for face photo-comparison has been praised by some [252], others have warned of errors, especially around slow-frame image wipes and fades [251]. In part due to these problems, the FISWG has reported that morphological feature-by-feature comparison is probably the only viable/valid method [273]; but, as noted above, the validation data are thin, and the legitimacy of the comparisons in any single case often hinges upon multiple factors.

For morphoscopic assessment, analyst expertise is often cited/used as a justification for reaching conclusions [252,253]. But it is worth noting “specialist knowledge” has been questioned in the courtroom since the contribution of “training, study or experience” to morphological comparison of photographs by anatomists/anthropologists has not been clearly elucidated [253,261]. It should also be noted that the sub-classifications provided by morphological feature atlases do not enable people to be identified (e.g. 19%–39% mismatching samples have been found when feature atlases were used in this fashion [269]).

Compared with morphometric and morphological analyses in 2D, 2D facial image evaluation using 3D physiognomic data [254–257] is less subjective and delivers promising results within certain limits [217–220]. Yoshino et al. [257] have tested this system using 16 comparative landmarks, 25 “matching” participants (250 superimpositions), and 24 participants who served as non-matches for each of the 25 participants mentioned above (24 × 25 comparisons =600 superimpositions). While these samples are not extensive in size, the results indicated that superimpositions of the same subject yielded mean landmark differences of 1.4 –3.3 mm whereas those of different subjects provided mean distances of 2.3 –4.7 mm [257]. The percentage error at the cross-over between false positives and true negatives (2.5 mm) was 4.2% [257], but the total percentage error was not specified/reported for any mean difference above 2.3 mm (the threshold of incorrect results). More recently, the discriminatory power of the system, and the impact of age, facial expression, and shared DNA (i.e. nine pairs of monozygotic twins) have been investigated from the frontal view only, with the results showing that the mean landmark differences provided a distance measure of 2.0 mm for twins (11 points) and thus failed to individuate in this context [263]. Significant differences in facial emotions are also problematic for the system, but change with age reportedly has a smaller impact [263].

A study with a small sample (*n* = 2), using a similar 2D-to-3D comparison approach to that employed by Yoshino et al. [257], concluded (using 11 comparison landmarks) that subjects could not be differentiated using the 2D-to-3D comparative approach [259]. While 2D-to-3D comparisons have been criticised for the quality of their 3D scans as a potentially problematic factor [253,261], either based on artefacts reported for older scanners [274] or time delay during scan acquisition (Schofield and Goodwin 2004 cited in [261]), it should be noted that the scanners referenced in prior work are now relatively old and were not the ones employed by the Japanese team (i.e. Fiore® range finding system by NEC®) [257]. Furthermore, commercial 3D scan technology continues to improve (see the “Facial approximation” section). While it is not perfect [282], some of the latest stereo-photogrammetry devices enable instantaneous capture, so that movement of the subject during data acquisition can be entirely avoided, and inaccuracy in capture is manageable when quality assurance regimes are employed in controlled environments (e.g. <0.5 mm of error [283–285]). In this context, it is more likely that the human error in placing landmarks [278–281] exceeds the scan error in 2D-to-3D photo comparison.

## Molecular photofitting

*Principle of the method:* to estimate facial appearance from a person’s DNA [165].

*Purpose:* as for facial approximation and facial composites, this face graphic acts as a point of interest to focus public attention on details of the case to generate additional investigative leads. The ultimate aim of molecular photofitting is the generation of a face that can be correctly recognised as the person to whom the DNA belongs [162,163,165,166]. Given general preferences to analyse biological evidence this method has the potential to supplant or supplement eyewitness descriptions of offenders’ faces in cases where DNA can be retrieved. The method may also be used to supplement facial approximation methods in cases involving skeletal remains [169,238,286].

*Method:* regions or locations of DNA known to encode certain physical characteristics and facial form are sequenced to yield information concerning a person’s facial appearance [162,163,166,238,287]. A facial graphic is digitally synthesised in 3D computer space using relationships between genes and the morphology of the human face often using principle component descriptions of its variation, or some other optimised measure (e.g. bootstrapped response-based imputation modelling) [162,238].

*Brief history:* work in this field is relatively recent (conducted within the last 10 years). It started with the identification of DNA sequences encoding facial traits of interest, such as eye colour [167,168,170,288–291], skin colour [288–290, 292,293], hair colour [168,289], genetic ancestry [165,294] and sex [295]. Gene association studies, including dense SNP sampling [296], genome-wide association studies (GWAS), and linkage analysis [162,297], have generated this knowledge in conjunction with studies on various craniofacial disorders [286,298] and those using animal models [299–301].

*Recent research developments:* in addition to genes known to be associated with the physical characteristics outlined above (e.g. for eye colour: rs12913832 (*HERC2*), rs1800407 (*OCA2*), rs1393350 (*TYR*), rs12203592 (*IRF4*), rs12896399 (*SLC24A4*), and rs16891982 (*SLC45A2* (*MATP*) [166,167]), 20 craniofacial candidate genes have been recently identified [163], and further GWAS studies on 9 478 608 SNPs have identified many more SNPs (1932) across 38 distinct loci and genes that have reached genome-wide significance for face shape [162].

Using dense quasi-landmark face templates (e.g. 10 000 points) and facial segmentation with hierarchical spectral clustering to split the face into 63 segments, the association of the above-mentioned SNPs at 15 replicated loci have been elucidated (four completely new loci), with the majority of affected segments in the nose or the lower quadrant of the face/chin [162]. Accuracy studies concerning the performance of these methods have been common for single facial features, and are being expanded to more holistic whole-face prediction. Ethical considerations, comments, and debates around the use of DNA for forensic identification, especially concerning such powerful methods as are used to predict faces from DNA sequences, should be noted [166,302,303].

*Accuracy:* With regard to whole-face molecular photofitting, it is important to note that sex and ancestry have thus far been the major factors driving or enabling face estimates (individual genetic loci have failed to significantly improve the results [238,303]). Further, in terms of the ability of molecular photofitting methods to generate recognisable faces, there is a paucity of validation data; tests have been conducted on approximately five subjects in total using, for example, 24 SNPs across 20 genes [238,304]. This limited testing, both in terms of validation and extent of the genes and SNPs analysed, is not surprising given the preliminary and emerging stages of the research. The genetic determinants of human facial morphology are indeed complex, and this has led to warnings that molecular photofitting may be too aspirational to ever be sufficiently achieved [305].

Regarding the estimation of specific biological or facial features, the accuracy of established methods has been and continues to be documented. The most accurately predictable externally visible character is sex, but the specific accuracy depends on the genetic test method or a combination of methods employed, and the samples being analysed [166]. In terms of more specific face traits, red hair colour and blue/brown colours of the iris are regarded as accurately predictable from genes alone [166]. Approximately 70% accuracy has been recorded for red hair prediction [289] whereas positive predictive intervals of colours of the iris ranged from 66%–100% for blue eyes [167,169,290,306,307] and 70%–100% for brown eyes [167,169,290,306,307]. Typically positive predictive values for brown eyes were higher (> 85%) than for blue (> 75%), with a drastic reduction in the same statistic for the so-called intermediate eye colours [169]. Predictive models for skin colour are also being investigated, tested, and validated [288,308] and tests for other biological characteristics will presumably follow.

## Discussion

In the forensic context, any method that draws on facial images may be collectively referred to as “facial imaging”. Largely underpinned by advances in computer processing power that facilitate numerical analysis and more detailed investigations of anatomical structure, progress in facial imaging methods over the past 20 years has been unprecedented. In some cases, it has made possible what was entirely unimaginable 20 years ago, e.g. somewhat reliable facial approximation methods where facial features unrelated to the skull can be estimated with reasonable degrees of accuracy using scientifically derived approaches. Challenges persist in the field, and much validation work needs to be undertaken, but the magnitude and value of recent achievements in the facial imaging sphere clearly count as among the more impressive in forensic anthropology and, more generally, have been made possible from interactions among diverse scientific disciplines. Not only do these advancements broaden the scope of what has previously been thought applicable in forensic anthropology (refer to the few facial imaging techniques discussed in key forensic anthropology texts [6,46,309–312]), but they have also rendered previous craniofacial identification methods more objective and, thus, brought them closer to the status of emerging scientific endeavours.
